# Analysis of Transient Thermoacoustic Characteristics and Performance in Carbon Nanotube Sponge Underwater Transducers

**DOI:** 10.3390/nano14100817

**Published:** 2024-05-07

**Authors:** Qianshou Qi, Zhe Li, Huilin Yin, Yanxia Feng, Zhenhuan Zhou, Dalun Rong

**Affiliations:** 1 State Key Laboratory of Structure Analysis of Industrial Equipment, Department of Engineering Mechanics, International Research Center for Computational Mechanics, Dalian University of Technology, Dalian 116024, China; qiqs@mail.dlut.edu.cn (Q.Q.); zheli20242024@gmail.com (Z.L.); huilinyin33@gmail.com (H.Y.); zhouzh@dlut.edu.cn (Z.Z.); 2 Jiangxi Copper Technology Institute Co., Ltd., Nanchang 330096, China; 19870088815@163.com; 3 School of Aeronautics and Astronautics, Shenzhen Campus of Sun Yat-sen University, Shenzhen 518107, China; 4 School of Civil Engineering, Hunan University of Technology, Zhuzhou 412007, China

**Keywords:** transient thermoacoustic analysis, CNT sponge, underwater transducer, thermoacoustic response time

## Abstract

Recent advancements in marine technology have highlighted the urgent need for enhanced underwater acoustic applications, from sonar detection to communication and noise cancellation, driving the pursuit of innovative transducer technologies. In this paper, a new underwater thermoacoustic (TA) transducer made from carbon nanotube (CNT) sponge is designed to achieve wide bandwidth, high energy conversion efficiency, simple structure, good transient response, and stable sound response, utilizing the TA effect through electro-thermal modulation. The transducer has potential application in underwater acoustic communication. An electro-thermal-acoustic coupled simulation for the open model, sandwich model, and encapsulated model is presented to analyze the transient behaviors of CNT sponge TA transducers in liquid environments. The effects of key design parameters on the acoustic performances of both systems are revealed. The results demonstrate that a short pulse excitation with a low duty cycle could greatly improve the heat dissipation of the encapsulated transducer, especially when the thermoacoustic response time becomes comparable to thermal relaxation time.

## 1. Introduction

Recent advancements in underwater acoustics highlight the critical role of ultrasonic transducers in enhancing the versatility and efficiency of marine applications [1]. Broadband underwater ultrasonic transducers have become essential for significant breakthroughs in fields of sonar detection [2], underwater communication [3], active noise cancellation systems [4], etc. Their wide frequency range enables more precise acoustic imaging, reliable communication, and effective noise reduction in challenging underwater environments. However, the commonly used piezoelectric transducers face limitations of their narrow frequency–response and signal crosstalk due to their resonant mechanism, which reduces precision in underwater detection [5] and efficiency in communication [6], and impedes effective noise suppression in active noise cancellation against the varied underwater soundscape [7,8]. Thus, there is a clear need to develop broadband, structurally simple, and flexible underwater transducers to address these limitations [9,10].

In recent days, a type of newly developed nanomaterial transducer [11,12,13,14,15,16,17,18,19,20,21,22,23,24,25,26,27,28] offers a promising solution by using the thermoacoustic (TA) effect [29]. TA transducers made of those nanomaterials operate by exchanging the produced Joule heating to the surrounding fluid and generating acoustic waves. This enables a broad frequency–response (1 Hz–20 MHz [30]), simpler design, and greater flexibility, avoiding the mechanical constraints of conventional devices. At the current stage, the existing research was concentrated on the airborne TA effects of carbon nanomaterials due to their exceptional thermal conductivity and low heat capacity [12,31,32,33,34]. Recent scholarly work [35,36,37,38] has explored the use of CNT or other nanomaterials for their piezoresistive properties, which could potentially be employed in acoustic receiving.

Compared to their airborne counterparts, underwater applications present more complex challenges that require TA transducers to be not only robust against liquid environments but also efficient in acoustic energy output. Addressing these requirements, Aliev et al. [34] investigated the TA performance of encapsulated underwater TA transducers made of CNT films, which generate wide-range, smooth-spectrum sound in water. A short-pulse excitation was employed to enhance efficiency through cooling during low-duty cycles and non-equilibrium TA processes. However, the two-dimensional (2D) CNT film TA transducers encounter significant obstacles: (1) they are easily damaged and unsuitable in a liquid environment due to their low mechanical strength through-thickness [34]; (2) they are unsuitable for high-intensity pulse excitation due to the quick heating up and slow heat dissipation rate of low-surface-area CNT film TA transducers [39]. The three-dimensional (3D) CNT sponge with porous structure attracted much interest due to its excellent self-supporting performance [40], high heat dissipation [39], and high mechanical stability [41]. Aliev et al. [39] measured the acoustic characteristics and bending ability of the CNT sponge, and they found that it had better heat transfer capacity and mechanical properties comparable to MWNT sheets. Guiraud et al. [42,43] presented a two-temperature model to describe the TA generation of 3D porous thermophones. Zhou et al. [44] developed a flexible and hi-fi CNT sponge loudspeaker that can exhibit a wide bandwidth, high-quality audio output, and stable sound response.

Despite the potential of CNT sponge transducers for underwater applications, the complexities of environment interaction and modeling have limited comprehensive experimental and theoretical studies. The TA simulation offers a promising solution to these challenges. However, existing simulations [45,46,47] have been confined to the frequency domain and cannot deal with transient behaviors associated with pulse excitation. To overcome these limitations, a comprehensive electro-thermal-acoustic coupled transient simulation capable of pulse excitation is introduced to design the underwater carbon nanotube sponge TA transducers for potential application in underwater acoustic communication. Firstly, the COMSOL Multiphysics software was used to establish the simulation models for underwater TA transducers. Subsequently, the influences of liquid medium, model structure size, and encapsulated gas on their thermoacoustic responses are examined. Finally, the heat dissipation conditions and the transient thermoacoustic field generated by the encapsulated transducer under short pulse excitation are explored, and the ways to improve the heat dissipation and sound pressure are discussed.

## 2. Model Designs of 3D CNT Sponge TA Transducers

**Figure 1:**
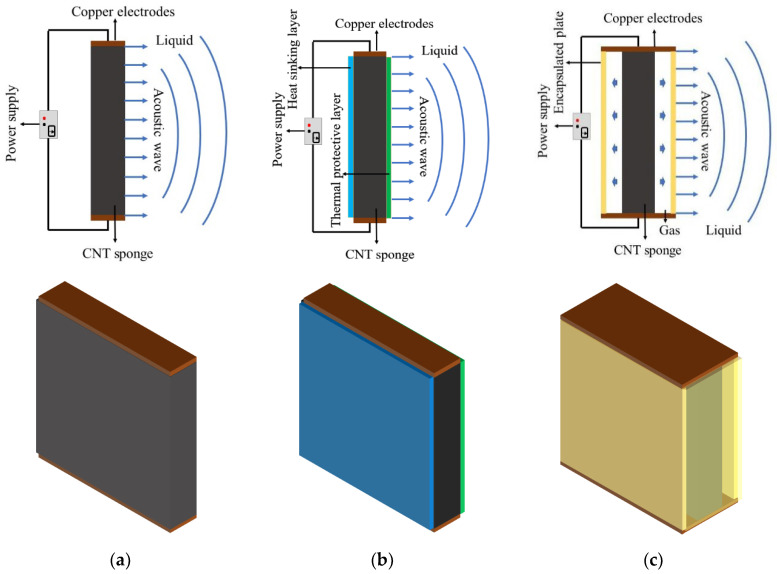
Schematic illustration and top view of three structures: (**a**) open model; (**b**) sandwich model; (**c**) encapsulated model.

To explore the effects of the different thermodynamic systems on acoustic properties, three TA transducers were designed: the open model, the sandwich model, and the encapsulated model. The schematic diagram and top view of these models are shown in Figure 1. The details of each model are as follows:

(i) Open model: A CNT sponge is connected to two copper electrodes, directly exposed to the liquid medium. This design allows for direct heat exchange with the external environment, facilitating the study of the TA effect in an unrestricted setting.
(ii) Sandwich model: A layered configuration consists of a thermal insulation layer (made of polydimethylsiloxane, PDMS), a core of CNT sponge with two copper electrodes, and a thermal dissipation layer (the heat-sink of graphene on copper foil substrate, Gr/Cu). The heat-sink of graphene on copper foil substrate (Gr/Cu) was chosen as the heat-sinking layer because of its high-temperature resistance and excellent thermal conductivity [48]. This structure is intended to optimize the transducer’s performance by enhancing thermal management and TA efficiency.
(iii) Encapsulated model: This model involves enclosing a CNT sponge that is enclosed within a polymethyl methacrylate (PMMA) plate casing, surrounded by encapsulated gas. This design protects the CNT sponge from direct contact with external liquid and improves TA performance using encapsulated gas.

The TA effect involves a time-varying thermal source, typically nanomaterials activated by electro-thermal modulation, to generate Joule heating, which induces heat exchange with the surrounding medium. This interaction creates a dynamic temperature gradient in the surrounding medium, thus emitting sound without relying on CNT sponge vibrations. In the three models, the CNT sponge connected to two copper electrodes with electrical excitations serves as the time-varying thermal source. For the open and sandwich models, the TA conversion occurs between the model and the external surrounding liquid medium; for the encapsulated model, the TA process takes place within its chamber with encapsulated gas, generating sound pressure and propagating it outside the structure.

The design parameters of the three models are tabulated in Table 1. To avoid strong thermal interaction, the thickness of the gas layer for the encapsulated model was set to 1 mm, which is greater than the thermal diffusion length of the gas [34].

## 3. Simulation Method

In this section, the TA performance of the three underwater CNT sponge TA transducers is investigated by using COMSOL Multiphysics software. The simulation consists of two steps:

(i) Analysis of Joule heating effect.

The Joule heating effect involves the coupling of electrical and thermal physics. In this paper, the electric current and fluid–solid heat transfer modules in COMSOL Multiphysics software are used to simulate the temperature fluctuations of the response excitation. There are two governing equations.

Transient electric current conservation:

(1) $$\nabla \cdot [\sigma (-\nabla V)+\frac{\partial D}{\partial t}+J_{e}]=Q_{j,v},$$

where $Q_{j,v}$ is the current source (A/m^3^), σ is the electrical conductivity (S/m), D is the electric displacement field (C/m^2^), $J_{e}$ is the external current density (A/m^2^), V is the electric potential (V). This equation follows Ohm’s Law, ensuring electric current conservation within the medium.

Heat transfer with convection:

(2) $$\rho C_{P}\frac{\partial T}{\partial t}+\rho C_{P}u\cdot \nabla T+\nabla \cdot \left(-k\nabla T\right)=Q,$$

where ρ is the density of the material (kg/m^3^), $C_{P}$ is the heat capacity at constant pressure (J/kg·K), T is the temperature (K), u is the velocity vector (m/s), Q is the internal heat source term (W/m^3^), and k is the thermal conductivity (W/m·K). This equation accounts for the transient temperature variations within the structure, incorporating thermal accumulation and convective heat transfer. $\rho C_{P}\frac{\partial T}{\partial t}$ is the thermal accumulation term, representing the transient temperature variation in the structure; $\rho C_{P}$ is the material’s heat capacity term; $\rho C_{P}u\cdot \nabla T$ is the thermal convection term involving fluids; ∇·(−k∇T) is the heat conduction term.

In addition, to address the computational demands of high-frequency, low-duty cycle pulse excitation, the events module is employed for precise timing of excitation changes, essential for accurate modeling under short pulse conditions.

(ii) Analysis of TA effects.

Based on the temperature distribution established by the Joule heating effect, the thermoviscous module simulates the sound pressure generated by the rapid thermal diffusion from the CNT sponge. The pressure acoustic module then works in conjunction with the thermoviscous module to simulate the propagation of the sound pressure field that propagates to the outside. There are three basic governing equations for the thermoviscous acoustic module.

Continuity equation for fluid motion:

(3) $$\frac{\partial \rho_{t}}{\partial t}+\nabla \cdot \left(\rho_{0}u_{t}\right)=0,$$

Navier–Stokes equation for momentum conservation:

(4) $$\nabla \cdot \left(-p_{t}I+\mu \left(\nabla \cdot u_{t}+{\left(\nabla \cdot u_{t}\right)}^{T}\right)-\left(\frac{2}{3}\mu -\mu_{B}\right)\left(\nabla \cdot u_{t}\right)I\right)=\rho_{0}\frac{\partial u_{t}}{\partial t},$$

General heat equation for energy conservation:

(5) $$\rho_{0}C_{P}\left(\frac{\partial T_{t}}{\partial t}+u_{t}\cdot \nabla T_{0}\right)-\alpha_{p}T_{0}\left(\frac{\partial p_{t}}{\partial t}+u_{t}\cdot \nabla p_{0}\right)=\nabla \cdot \left(k\nabla T_{t}\right)+Q,$$

where μ is the dynamic viscosity (Pa·s), $\mu_{B}$ is the bulk viscosity (Pa·s), k is the thermal conductivity (W/(m·K)), $C_{P}$ is the constant pressure heat capacity (J/(kg·K)), $\alpha_{p}$ is the thermal expansion coefficient (m^2^/s), I is the unit matrix, $\rho_{t}$ is the density (kg/m^3^), $u_{t}$ is the velocity vector (m/s), $p_{t}$ is the sound pressure (Pa), $T_{t}$ is the change in temperature (K), $\rho_{0}$ is the equilibrium density (kg/m^3^), $p_{0}$ is the equilibrium sound pressure (Pa), and $T_{0}$ is the equilibrium temperature (K). Equations (3)–(5) explicitly describe the propagation of sound waves that include thermal and viscous losses. They are known as thermo-viscous acoustic equations, and the associated problems are also referred to as thermo-viscous acoustics.

The governing equation of the pressure acoustic module is

(6) $$\frac{1}{\rho c^{2}}\frac{\partial^{2}p_{t}}{\partial t^{2}}+\nabla \cdot \left(-\frac{1}{\rho }\left(\nabla p_{t}-q_{d}\right)\right)=Q_{m},$$

where $q_{d}$ is the dipole source (N/m^3^), $Q_{m}$ is the unipolar source (1/s^2^), ρ is the fluid density (kg/m^3^), and c is the sound velocity in the fluid region (m/s).

The interface continuity conditions of the two computational regions are

(7) $$-n\cdot \left(-\frac{1}{\rho_{c}}\left(\nabla p_{t}-q_{d}\right)\right)=-n\cdot \frac{\partial u_{t}}{\partial t},$$

(8) $$\left(-p_{t}I+\mu \left(\nabla \cdot u_{t}+{\left(\nabla \cdot u_{t}\right)}^{T}\right)-\left(\frac{2}{3}\mu -\mu_{B}\right)\left(\nabla \cdot u_{t}\right)I\right)n=-p_{t}n,$$

(9) $$-n\cdot \left(-k\nabla T_{t}\right)=0,$$

where n is the unit normal vector of the interface.

The boundary conditions of the CNT sponge TA transducer are determined by the amplitude of the temperature change and the pressure gradient on the surface of the sponge,

(10) $$\frac{\partial p_{t}}{\partial n}=0,$$

(11) $$T_{t}=\hat{T_{f}},$$

where $\hat{T_{f}}$ is the amplitude of the temperature oscillation on the surface of the CNT sponge (K).

Finally, the perfect matching layer (PML) is selected as the absorption boundary to truncate the calculation region, and the absorption boundary absorbs the sound wave transmitted to the boundary without reflection.

This above-integrated simulation analysis provides a different insight into the TA dynamics of underwater CNT sponge TA transducers, offering a detailed perspective on their behavior under various excitations.

## 4. Results and Discussion

### 4.1. Comparison Studies with Experimental and Theoretical Results

**Figure 2:**
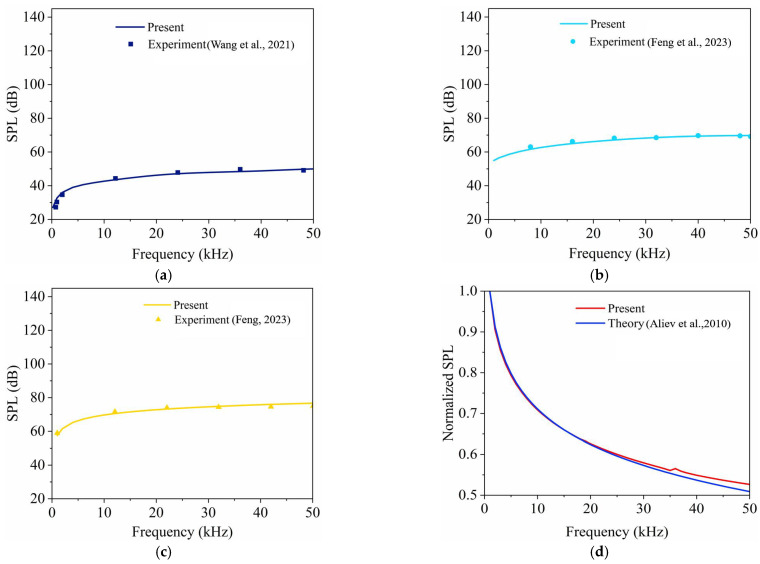
Comparison studies of the three models: (**a**,**b**) open model in air (Wang et al., 2021 [44]) and water (Feng et al., 2023 [49]); (**c**) sandwich model in water (Feng, 2023 [51]); (**d**) encapsulated model in water (Aliev et al., 2023 [34]).

In this section, the frequency–responses of three models of underwater TA transducers are presented in Figure 2. Comprehensive details on the physical and thermal properties of materials are shown in Table 2, taken from references [44,49,50].

In Figure 2a,b, we present a detailed comparative analysis of the thermoacoustic behavior of open CNT sponge in both air and water, respectively. The simulation results for the open CNT sponge in air were compared with the experimental findings reported by Wang et al. [44], and those in water were compared with the findings of Feng [51]. The comparisons are favorable, with the maximum error recorded being 2.037 dB in Figure 2a and 2.176 dB in Figure 2b. In other words, the proposed open model is appropriate for use with both air and water. However, this model is not appropriate for liquids such as ethanol due to the affinity of the CNT sponge for these media [34]. This issue is further discussed in Section 4.2.

Regarding the sandwich model, since there are no available experimental results for sandwich CNT sponges, a sandwich CNT film model is developed. In Figure 2c, the simulation results are compared with the experimental results of a sandwich CNT film [49] using the same simulation method. The maximum error is 2.466 dB. This small discrepancy further confirms the accuracy of the proposed simulation method for predicting the underwater TA behavior of the sandwich model despite the absence of direct sponge model validation.

In addition, due to the lack of available comparison for encapsulated CNT sponges, an encapsulated CNT film model is simulated, as shown in Figure 2d. The sound pressure level (SPL) of the encapsulated model shows a gradually decreasing trend as the frequency increases. This trend is consistent with theoretical predictions for an encapsulated CNT film transducer [34]. This supports the validity of our proposed simulation approach for predicting the behavior of the encapsulated model.

In summary, these comparison studies demonstrate that our proposed simulation method could accurately predict the TA behavior of CNT sponge underwater TA transducers.

### 4.2. Models within Different Liquids

**Figure 3:**
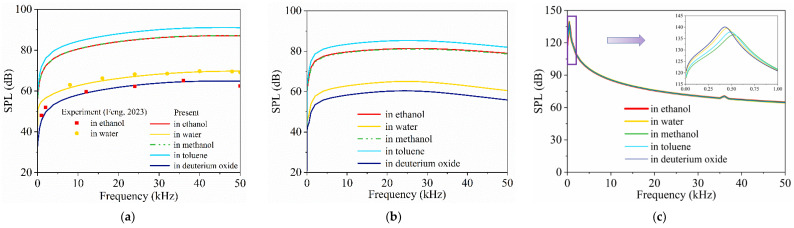
Acoustic pressure response of the CNT sponge transducer within different liquids: (**a**) open model (Feng, 2023 [51]); (**b**) sandwich model; (**c**) encapsulated model.

In this section, the frequency–response of three models of underwater CNT sponge TA transducers in different liquid environments (water, methanol, ethanol, toluene, and deuterium oxide) are investigated. The properties of these liquids are presented in Table 3. The acoustic pressure response of the three models is depicted in Figure 3.

For the open model, as shown in Figure 3a, the simulation results of SPL in water closely match experimental results [51], where tests were conducted on an open model made of CNT sponge in both water and ethanol. However, the difference between the simulation and experimental results in ethanol [51] is significant. This discrepancy is due to the high hydrophobicity of the CNT sponge, which creates a gas envelope around its surface [52]. In contrast, when ethanol contacts the CNT sponge directly, it increases the thermal inertia of the sponge and leads to a reduction in the SPL in ethanol [53]. Therefore, the SPL from the open CNT sponge significantly decreases if the CNT sponge has an affinity for the liquid medium. It should be mentioned that the present open model is not appropriate for liquids such as ethanol. This is due to the fact that the present model cannot capture the effects of interface interaction if the CNT sponge has an affinity for this medium. Furthermore, the open CNT sponge TA transducer may experience a short-circuit fault and induce electrochemical reactions in seawater, given its ionic conductor [34]. Given these potential issues with external liquid interaction, the open model is not ideal for underwater applications.

**Figure 4:**
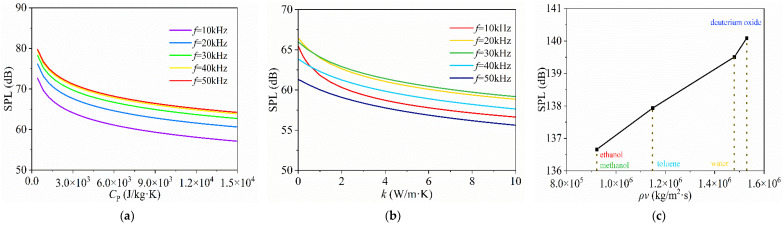
Acoustic pressure response of the sandwich or encapsulated CNT sponge transducers with different properties of liquids: (**a**) SPLs versus specific heat capacity of the liquid medium of the sandwich model; (**b**) SPLs versus thermal conductivity of liquid medium of the sandwich model; (**c**) peak SPL versus sound velocity and density of the encapsulated model.

In Figure 3b, the sandwich model presents the highest SPL in toluene and the lowest SPL in deuterium oxide. Further analyses in Figure 4a,b show that the sandwich model’s SPL decreases with the increasing specific heat capacity and thermal conductivity of liquid medium, indicating the significant impact of heat loss to liquid medium on TA conversion efficiency [34]. Therefore, toluene/deuterium oxide can produce the highest/lowest SPL of the above five liquids due to its low/high specific heat capacity and high thermal conductivity, respectively.

The encapsulated model maintains consistent SPLs across these liquids, as shown in Figure 3c. This consistency indicates that the encapsulated model’s TA efficiency, mainly determined by its encapsulated gases, is less sensitive to the external liquid’s thermal properties. Figure 4c shows that the encapsulated model’s SPL is in direct proportion to acoustic impedance, but the slope is not high. This explains the phenomenon observed in Figure 3c, where the SPLs across different media depend on their acoustic impedance, and the difference is not large. It indicates that the acoustic impedance of the liquid affects the sound propagation of the transducer but does not significantly influence the TA conversion efficiency in the encapsulated model.

From the analysis above, the open model and the sandwich model are significantly influenced by external liquids. In contrast, the encapsulated model’s designs effectively protect its TA process from liquid interaction, enhancing its adaptability for underwater environments.

### 4.3. Sandwich and Encapsulated Models with Different Thicknesses

In this section, the influence of structural thickness variations on the TA outputs of the sandwich model and the encapsulated model are investigated. Figure 4 investigates the impact of the layer thicknesses of the encapsulating structure, CNT sponge, and thermal layers on the TA effects.

**Figure 5:**
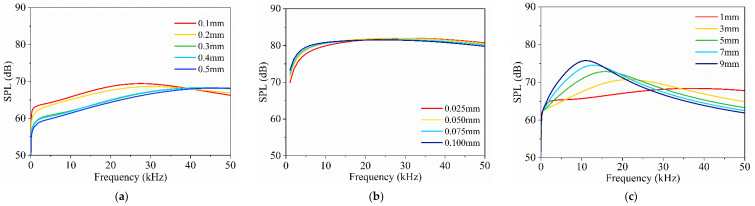
Effect of different structural thickness parameters on TA frequency–response of the sandwich model: (**a**) thickness of heat sinking layer; (**b**) thickness of protective layer; (**c**) thickness of CNT sponge.

For the sandwich model, the effects of thickness variations in the heat-sinking layer, protective layer, and CNT sponge are examined in Figure 5. Increasing the thickness of the heat-sinking layer results in a decrease in SPL at low frequency (<40 kHz) and an increase at high frequency (>40 kHz). This suggests that a thicker heat-sinking layer reduces the temperature gradient during low-frequency excitation but improves the TA conversion efficiency in high-frequency heat exchangers. In contrast, a thicker protective layer and CNT sponge enhance SPL at low frequency but reduce SPL at high frequency. This suggests that a thinner protective layer and CNT sponge decrease the heat capacity of the systems, making them more efficient for high-frequency applications. The optimal thicknesses of the heat-sinking layer depend on the output frequency.

**Figure 6:**
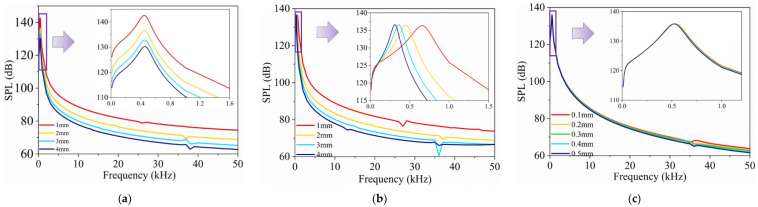
Effect of different structural thickness parameters on TA frequency–response of the encapsulated model: (**a**) thickness of CNT sponge; (**b**) thickness of encapsulated gas layer; (**c**) casing thickness.

For the encapsulated model, Figure 6 presents the SPL frequency–response curves for different thicknesses of the CNT sponge, encapsulated gas layer, and PMMA plate casing. The response curves show resonance and anti-resonance peaks, along with a negative slope at higher frequencies. This indicates the interaction between TA effects and the model’s structural dynamics, which leads to a notable SPL enhancement at the lower end of the frequency spectrum. As shown in Figure 6a,b, the SPL decreases with increased thickness of the CNT sponge and encapsulated gas layer due to the larger volume, which leads to a reduced power density of TA conversion. Moreover, the heat capacity per unit area of the CNT sponge increases with thickness [44], making heat dissipation more challenging and resulting in a lower SPL. However, the thickness of the PMMA plate casing has little influence on the SPL of the model, as shown in Figure 6c.

Generally, a thinner encapsulated model provides a more consistent and higher SPL response, even with a very thin encapsulated gas layer. Moreover, the encapsulated model produces significantly higher SPL than the sandwich model due to the coupled effects of TA and structural dynamics, especially at low frequencies.

### 4.4. Effects of Encapsulated Gases on TA Emitting of the Encapsulated Model

**Figure 7:**
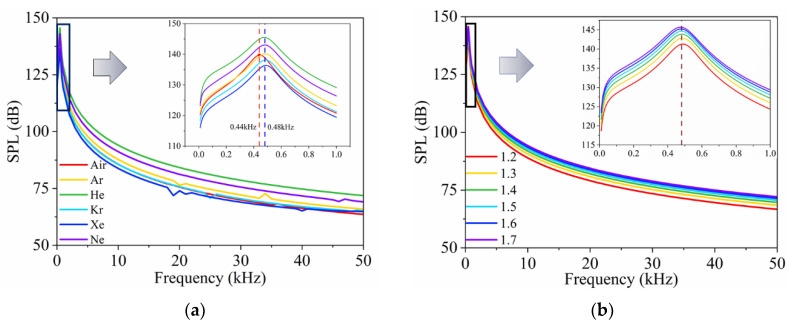

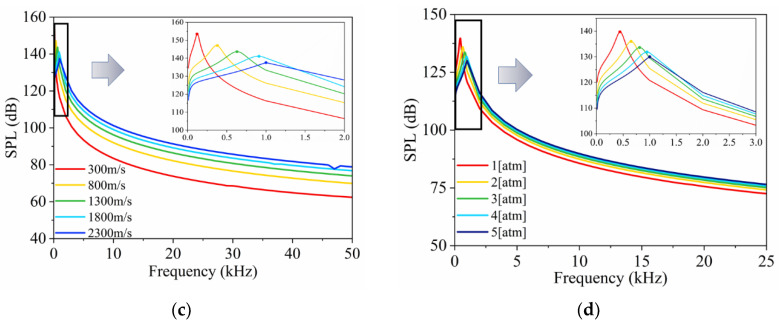
Frequency–response of SPL of physical and thermal property parameters of the encapsulated gas in the encapsulated model: (**a**) various encapsulated gases; (**b**) variation in specific heat ratio; (**c**) variation in speed of sound; (**d**) variation in gas pressure.

The investigations in Figure 4c established that the SPL produced by an encapsulated CNT sponge TA transducer is less sensitive to the external liquid medium. This section extends this examination by considering the physical and thermal properties of different gases within the encapsulated structure and how these properties influence the TA effects, as shown in Figure 7.

Figure 7a presents the SPL response of the encapsulated transducer filled with different gases, including air, argon (Ar), helium (He), krypton (Kr), xenon (Xe), and neon (Ne). The properties of these gases are detailed in Table 4. Helium (He) provides the superior SPL response, whereas xenon (Xe) yields the lowest SPL values.

The difference in SPL performance among these gases is further analyzed in Figure 7b–d. Figure 7b illustrates the influence of varying specific heat ratios (γ values of 1.2–1.7) on the SPL response. The data indicate that higher specific heat ratio values lead to a stronger SPL. However, the specific heat ratios negligibly affect the peak frequency of the SPL response. Figure 7c examines the impact of different speeds of sound on the SPL response. The results reveal that lower speeds of sound (*v* = 300, 800, 1300, 1800, 2300 m/s) result in higher SPL peaks and lower peak frequencies. This indicates that slower sound speeds within the encapsulated gas enhance the peak SPL and shift the response to lower frequencies. Figure 7d demonstrates the effect of internal gas pressure variations on the TA response. Higher pressure moves the response curve to higher frequencies and lowers the SPL peak. Moreover, increased pressure conditions favor SPL performance at higher frequencies.

In summary, the strategic selection of encapsulated gases, such as helium, significantly improves the SPL performance of encapsulated models by balancing key physical properties like specific heat ratio and speed of sound.

### 4.5. Enhancement of Encapsulated TA Transducer Performance with Short Pulses Excitation

From the above analysis, it was found that the encapsulated TA transducer with an inert gas can enhance sound pressure. However, a closed chamber interior results in limited heat dissipation, which increases the overall temperature and prevents further improvements in the transducer’s performance [33]. To address these challenges, this section examines the application of short pulses to drive the encapsulated TA transducer.

#### 4.5.1. Temperature Distributions and Heat Dissipation Conditions

**Figure 8:**
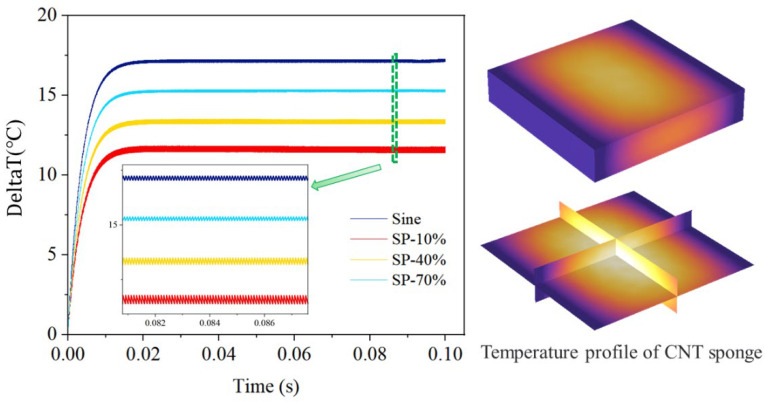
The surface temperature of the CNT sponge and the overall temperature distribution of the encapsulated model for four excitations: sinusoidal excitation; excitations with SP-10%, SP-40%, and SP-70%.

Figure 8 shows the surface temperature of the CNT sponge and the overall temperature distribution within the encapsulated model under four types of excitations: sinusoidal excitation; short pulse excitations with a duty cycle of 10%, 40%, and 70% (denoted as SP-10%, SP-40%, and SP-70%, respectively). At a *P*_in_ = 1 W and *f* = 50 kHz, excitation with a low duty cycle results in a lower stable temperature, larger temperature fluctuation after equilibrium, and a pronounced overall temperature gradient across the device. This increase is due to reduced cooling time for the heat source and encapsulated gas to reach ambient temperature as the duty cycle increases [33]. These findings suggest that short pulse excitation with low-duty cycles can significantly enhance heat dissipation in the device, which suggests a potentially longer lifespan than the data measured in Wang’s experiment [44] under comparable power settings.

A theoretical solution can be used for reference by the following expression [33]:

(12) $$\frac{P_{\mathrm{in}}}{S}=\rho C\frac{dT}{dt},$$

where *T* and *t* are the temperature after pulse excitation and the corresponding pulse width, respectively; *C* is the gravimetric specific heat capacity of the CNT sponge.

#### 4.5.2. Transient Sound Response Performance of Encapsulated TA Transducers under Short Pulses Excitation

**Figure 9:**
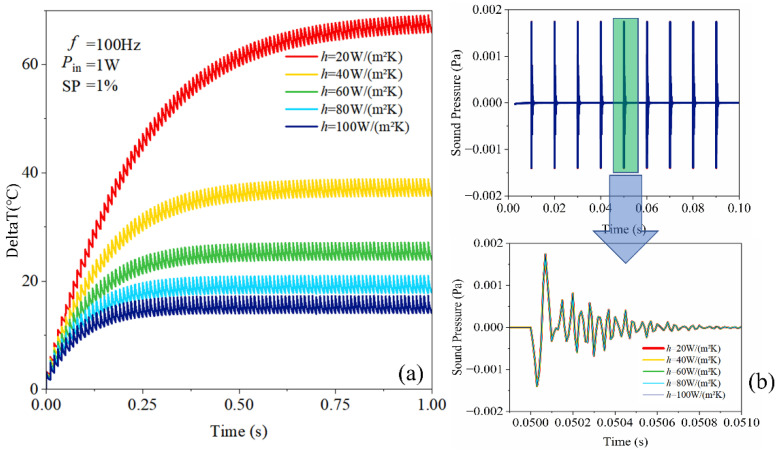
The correlation between the transient response and the heat transfer coefficient of CNT sponge: (**a**) temperature response; (**b**) sound pressure response.

The transient sound response performance of encapsulated TA transducer for short pulse excitation is investigated in this section. An electro-thermal simulation analyzed how the heat transfer coefficient affects the CNT sponge’s transient TA response with *P*_in_ = 1 W, SP-1%, and *f* = 100 Hz, as shown in Figure 9. Figure 9a shows that, as the heat transfer capacity of TA materials increases, the system reaches its steady-state temperature more quickly and maintains a lower average while the amplitude of temperature fluctuation remains unchanged.

Based on this temperature response, a thermoacoustic analysis within the time domain was conducted, as depicted in Figure 9b, indicating that different heat transfer coefficients yield identical and stable sound pressure responses. The sound pressure demonstrates a sharp increase and decrease in waveform during a short pulse. This behavior results from the expansion and contraction of the ambient medium at the pulse’s rising and falling edges, respectively [54]. This finding illustrates the CNT sponge TA transducer’s rapid transient response with short pulse excitation. It is noted that although a higher heat transfer coefficient lowers the stable average temperature of the system, it does not influence the amplitude of temperature oscillation caused by pulse excitation. Therefore, materials with high heat transfer coefficients enable low-temperature operation without compromising thermoacoustic efficiency.

**Figure 10:**
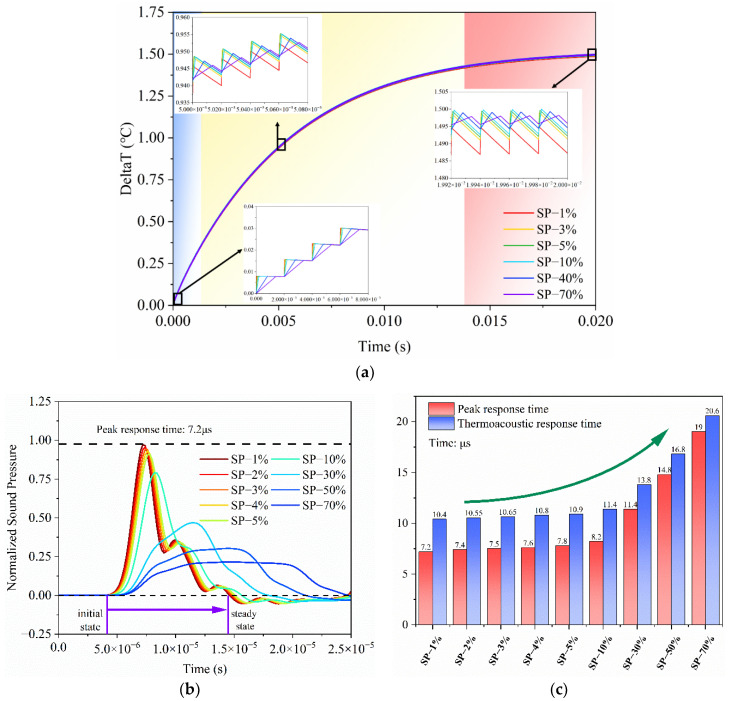
Transient response performance of the encapsulated model under different excitation: (**a**) electro-thermal response; (**b**) thermoacoustic response; (**c**) response time.

Furthermore, to demonstrate how thermoacoustic systems respond to varied excitations, electro-thermo-acoustic simulations were conducted, as shown in Figure 10. As the duty cycle of the short pulse decreases, the input energy becomes more concentrated over shorter intervals, altering the heat exchange between the material surface and the surrounding fluid. This leads to a noticeable reduction in the surrounding temperature and an increase in temperature fluctuations, thereby enhancing TA sound pressure and efficiency. Importantly, as the duty cycle falls below 1%, the electro-thermo-acoustic response of the system to pulse excitation reaches a threshold and then becomes saturated.

This threshold indicates that the thermal exchange mechanisms within the surrounding medium have finite limits governed by the medium’s heat capacity and thermal conductivity [33]. These properties limit the maximum rate at which heat can be dissipated from the nanomaterial, resulting in heat accumulation and a plateau in temperature variance and sound pressure when thermal transients exceed the medium’s diffusion capacity.

To further illustrate, the concept of thermal relaxation time, indicating how quickly the medium returns to equilibrium, is introduced and estimated using the formula $\tau =L^{2}/\alpha$, where L is the characteristic length of the heat transfer path, and α is the thermal diffusivity of the medium. Comparing this estimated thermal relaxation time of 6.369 μs with the thermoacoustic response time of 7.2 μs corresponding to the critical duty cycle of 1% offers solid evidence of the analysis. If the response time becomes comparable to or shorter than this relaxation time, the medium cannot return to baseline temperature between pulses, resulting in saturation. This analysis validates the observed saturation behavior, demonstrating the interaction between pulse excitation characteristics and the medium’s thermal properties.

To optimize thermoacoustic conversion efficiency, it is critical to match the temporal characteristics of the thermal pulse with the thermal response of the surrounding medium. This requires a comprehensive understanding of how the duty cycle of electrical excitation interacts with the thermal properties of both the nanomaterial and the medium.

Future research will explore the parameter near the saturation point to determine the optimal conditions for maximizing the acoustic output without exceeding the thermal exchange capabilities of the system, providing a pathway for optimization of the transient TA effect.

## 5. Conclusions

A novel underwater transducer composed of a 3D CNT sponge with a wide frequency range based on TA effects is designed and analyzed for potential application in underwater acoustic communication. Numerical simulation models of these transducers are developed to explore their acoustic properties. The effects of key design parameters for three thermodynamic systems on acoustic properties are investigated. Moreover, the heat dissipation conditions, energy conversion efficiency, and transient performance of the encapsulated transducer by short pulse excitations are investigated in detail. Numerical simulations reveal that the heat dissipation and acoustic performance of the encapsulated transducer are enhanced by using a short pulse excitation, especially when the thermoacoustic response time becomes comparable to thermal relaxation time. In conclusion, this work brings the possibility of developing wideband, high-efficiency, miniaturized, and low-cost underwater TA transducers for broadband applications.

## Figures and Tables

**Table 1:** Design parameters of the three models.

Model	CNT Sponge		Protective Layer	Heat-Sinking Layer	Gas Layer	Encapsulated Plate
	Area (mm^2^)	Thickness (mm)	Thickness (mm)	Thickness (mm)	Thickness (mm)	Thickness (mm)
Open model	10 × 10	2	/	/	/	/
Sandwich model	10 × 10	2	0.025	0.1	/	/
Encapsulated model	10 × 10	2	/	/	1	0.3

**Table 2:** The physical and thermal properties of materials (20 °C, 1[atm]).

Material	Cp (J/(kg·K))	ρ (kg/m^3^)	κ (W/(m·K))	Ref.
CNT sponge	500	10	0.021	[44]
CNT thin film	716	1390	0.0262	[49]
PDMS	1250	1000	0.15	[49]
Gr/Cu	540	8900	1500	[48]
PMMA	1464	1190	0.19	[50]

**Table 3:** Material properties of liquid environments (20 °C, 1[atm]).

Liquid	ρ (kg/m^3^)	v (m/s)	Cp (J/(kg·K))	γ (1)	κ (W/(m·K))	ρv (kg/m^2^·s)
Water	998.21	1482.3	4184.1	1.006	0.59801	1,479,646
Ethanol	789.47	1168.0	2431.4	1.193	0.16730	922,100
Methanol	791.01	1116.2	2504.6	1.204	0.20116	882,925
Toluene	866.89	1324.3	1685.4	1.351	0.13178	1,148,022
Deuterium oxide	1105.30	1384.0	4199.6	1.002	0.58865	1,529,735

**Table 4:** Material parameters of encapsulation gas (20 °C, 1[atm]).

Gas	ρ (kg/m^3^)	v (m/s)	Cp (J/(kg·K))	γ (1)	κ (W/(m·K))
Air	1.2044	343.16	1005.4	1.400	0.0262
Ar	1.6614	317.50	521.6	1.669	0.0174
He	0.1663	1007.90	5193.2	1.660	0.1493
Kr	3.4912	220.07	249.26	1.672	0.0093
Xe	5.4885	175.50	160.19	1.678	0.0054
Ne	0.8385	448.93	1030.4	1.667	0.0485

## Data Availability

Data are contained within the article.
